# Characteristics and outcome of traumatic cardiac arrest at a level 1 trauma centre over 10 years in Sweden

**DOI:** 10.1186/s13049-022-01039-9

**Published:** 2022-10-17

**Authors:** Daniel Ohlén, Magnus Hedberg, Paula Martinsson, Erik von Oelreich, Therese Djärv, Malin Jonsson Fagerlund

**Affiliations:** 1 Perioperative Medicine and Intensive Care, Karolinska University Hospital, Stockholm, Sweden; 2 Department of Physiology and Pharmacology, Section for Anaesthesiology and Intensive Care Medicine, Karolinska Institutet, Stockholm, Sweden; 3 Department of Acute and Reparative Medicine, Karolinska University Hospital, Stockholm, Sweden; 4 Department of Medicine, Karolinska Institutet, Stockholm, Sweden

**Keywords:** Traumatic cardiac arrest, Resuscitative thoracotomy, Resuscitation, Trauma, Outcome.

## Abstract

**Background:**

Historically, resuscitation in traumatic cardiac arrest (TCA) has been deemed futile. However, recent literature reports improved but varying survival. Current European guidelines emphasise the addressing of reversible aetiologies in TCA and propose that a resuscitative thoracotomy may be performed within 15 min from last sign of life. To improve clinician understanding of which patients benefit from resuscitative efforts we aimed to describe the characteristics and 30-day survival for traumatic cardiac arrest at a Swedish trauma centre with a particular focus on resuscitative thoracotomy.

**Methods:**

Retrospective cohort study of adult patients (≥ 15 years) with TCA managed at Karolinska University Hospital Solna between 2011 and 2020. Trauma demographics, intra-arrest factors, lab values and procedures were compared between survivors and non-survivors.

**Results:**

Among the 284 included patients the median age was 38 years, 82.2% were male and 60.5% were previously healthy. Blunt trauma was the dominant injury in 64.8% and median Injury Severity Score (ISS) was 38. For patients with a documented arrest rhythm, asystole was recorded in 39.2%, pulseless electric activity in 24.8% and a shockable rhythm in 6.8%. Thirty patients (10.6%) survived to 30 days with a Glasgow Outcome Scale score of 3 (n = 23) or 4 (n = 7). The most common causes of death were haemorrhagic shock (50.0%) and traumatic brain injury (25.5%). Survivors had a lower ISS (P < 0.001), more often had reactive pupils (P < 0.001) and a shockable rhythm (P = 0.04). In the subset of prehospital TCA, survivors less frequently received adrenaline (epinephrine) (P < 0.001) and in lower amounts (P = 0.02). Of patients that underwent resuscitative thoracotomy (n = 101), survivors (n = 12) had a shorter median time from last sign of life to thoracotomy (P = 0.03), however in four of these survivors the time exceeded 15 min.

**Conclusion:**

Survival after TCA is possible. Determining futility in TCA is difficult and this study demonstrates survivors outside of recent guidelines.

**Supplementary Information:**

The online version contains supplementary material available at 10.1186/s13049-022-01039-9.

## Introduction

Trauma claims around 5 million lives annually worldwide [1] and is the leading cause of death among young adults in industrialised countries [2]. Traumatic cardiac arrest (TCA) is the extreme state of traumatic shock and can be diagnosed when a patient, after suffering physical trauma, presents with both unconsciousness, agonal or absent spontaneous breathing and loss of a central pulse [3, 4]. Historically, resuscitative attempts in TCA were considered futile due to reported survival as poor as 0% [5]. However, more recent studies have shown improved albeit variable survival between 2.4 and 18.4% [3, 6–17]. In Sweden specifically, survival after out-of-hospital TCA gradually increased from 1.9 to 8.3% between 1990 and 2015 [3]. To further enhance care in TCA, the European Resuscitation Council (ERC) has developed a treatment algorithm stressing the importance of rapidly addressing reversible causes and suggests that emergency resuscitative thoracotomy may be performed in selected cases if less than 15 min have elapsed since loss of vital signs [4]. In TCA or peri-arrest the salvageability of resuscitative thoracotomy is quoted to be 7.8–21.8% depending on injury mechanism and setting [18–20], with only 6% reported to have neurological impairment [18]. For the trauma population in general it is also believed that care at designated trauma centres can reduce mortality [21, 22], especially in severely injured patients [23].

Given the dismal but not futile prognosis for TCA, it is important for the clinician to understand factors prognostic for survival and to identify whom may benefit from a resuscitative thoracotomy. The primary aim of this study was therefore to describe the characteristics and 30-day survival after TCA at a Swedish level 1 trauma centre with special regard to patients undergoing resuscitative thoracotomy.

## Methods

### Setting

Karolinska University Hospital Solna is the Swedish capital Stockholm’s only level 1 trauma centre serving a population of about 2.4 million and receives roughly 1400 adult trauma patients yearly [24]. From the trauma room, CT-scanners and operating theatres are immediately accessible, and a surgeon trained in performing a resuscitative thoracotomy is always part of the trauma team [25]. Local hospital protocols advise that resuscitative thoracotomy should be done within 10 min for blunt and 15 min for penetrating trauma. When applicable, aortic compression and internal cardiac massage is considered routine during thoracotomy. Resuscitative Endovascular Balloon Occlusion of the Aorta (REBOA) is not used for TCA.

All Emergency Medical Services (EMS)-crews in Sweden are trained in Advanced Cardiac Life Support (ACLS) and Prehospital Trauma Life Support (PHTLS). A physician staffed rapid response unit with advanced airway competency can be dispatched to assist the EMS in the Stockholm area. Prehospital thoracotomies are never performed in the region.

### Study design

A retrospective cohort study was conducted based on the Swedish Trauma Registry (SweTrau) and the Swedish Registry for Cardiopulmonary Resuscitation. Adult patients (≥ 15 years) with TCA managed at the Karolinska University Hospital Solna, Stockholm, Sweden from January 1st 2011 to December 31th 2020 were included. Patients aged < 15 years, invalid registry entries or medical records, cardiac arrest resulting from hanging, drowning, burns or smoke inhalation or who received bystander cardiopulmonary resuscitation (CPR) but with spontaneous circulation upon arrival of EMS were excluded. Patients exclusively attended by EMS and thus not transported to hospital were not studied. The decision to terminate resuscitation in the prehospital setting is made at the discretion of the treating physician or EMS-nurse. Patients with injuries incompatible with life or who did not respond to initial resuscitation with a predicted long transport time would be declared dead on scene.

For this survey, TCA was defined as comatose patient with absent or agonal breathing and no central pulse after a traumatic event. Prehospital TCAs were prespecified in SweTrau through the initiation of CPR either by bystanders or EMS crews. Thoracotomy was utilised as a proxy to identify patients who arrested in the initial hospital phase. All reports of thoracotomies in SweTrau were queried and patients included if they had a concurrent cardiac arrest. Patients in the Swedish Registry for Cardiopulmonary Resuscitation where EMS reported trauma as the presumed aetiology were also included.

### Data sources and variables

SweTrau is a nationwide registry of trauma patients. Patients are entered in the registry if a trauma call is triggered at the receiving hospital or if they are admitted with an Injury Severity Score (ISS) > 15. All trauma fatalities within 30 days at the Karolinska University Hospital also consistently undergo a structured review by a multidisciplinary committee and classified as preventable, possibly preventable or unpreventable [26].

The Swedish Registry for Cardiopulmonary Resuscitation is a national quality register for out of hospital cardiac arrest in Sweden. Patients are entered in the registry if EMS or bystanders attempted resuscitation.

Both registries report data according to standardised Utstein methodology [27].

Variables were collected from both SweTrau and through review of dispatch reports and digital hospital charts. Electronic records were scrutinized by two of the authors (DO, PM). Data concerning patient demographics, trauma mechanism, cause of death, vital signs, lab tests, performed interventions, EMS times, survival and functional outcome were extracted (Supplementary Table 1).

### Outcome

Our primary outcome measure was 30-day survival. Secondary outcome was functional status at discharge from the Karolinska University Hospital Solna expressed as Glasgow Outcome Scale (GOS) score. The scale contains five categories: (1) dead, (2) vegetative state, (3) severe disability, (4) mild disability and (5) good recovery [28]. Additionally, factors associated with survival were investigated in a sub-analysis.

### Statistics

Descriptive data were presented as median with interquartile range (IQR) for continuous variables and as numbers with percentages for categorical variables. Comparison between groups were made using Wilcoxon rank sum test for continuous variables and Chi-square test for dichotomous variables. Trends were evaluated using linear regression. Significance level was set to 0.05. Missing data were kept missing, i.e. not imputed or estimated. All data were analysed using R version 4.0.4 (R-studio core team 2021).

## Results

### Demography

**Fig. 1:**
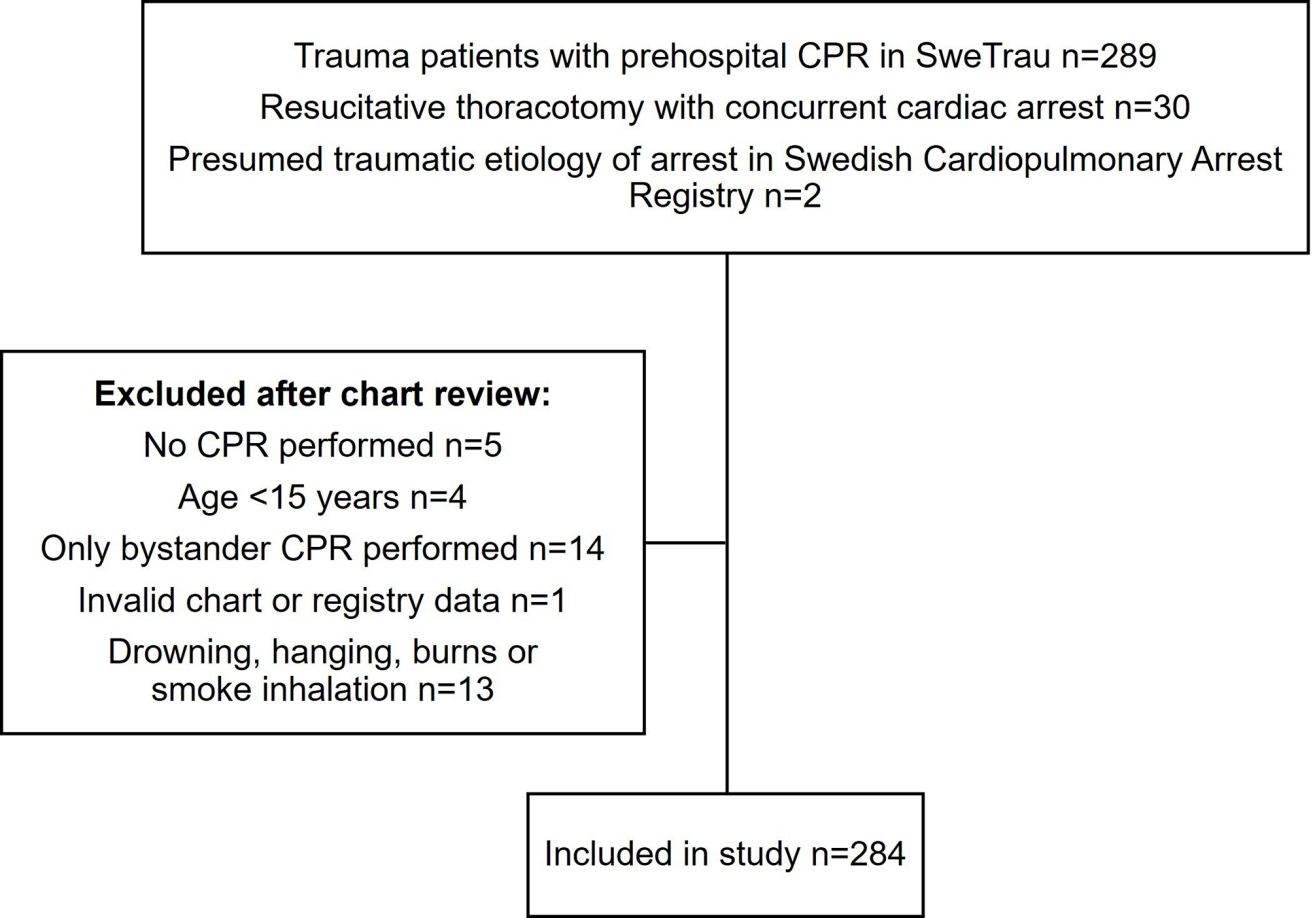
Flow chart of patients included in the study. CPR = cardiopulmonary resuscitation

In total, 284 patients with confirmed TCA were included in the study (Fig. 1). The median age was 38 years, 82.0% were male and 60% were previously healthy. The median ISS was 38. Cardiac arrest occurred out of hospital in most patients (90.1%). Blunt trauma was the predominant injury mechanism (64.8%). In patients with penetrating trauma 44.0% had suffered gunshot wounds and 56% stab wounds. For patients with a documented cardiac rhythm, asystole was observed in 39.4% and pulseless electric activity (PEA) in 24.6% whereas a shockable rhythm was recorded in 6.7% (Table 1). Among the 85 patients (29.9%) who survived to intensive care unit admission, the median length of hospital stay was 5 (IQR 3–20) days and the median time on ventilator was 3 (IQR 1–7) days.

**Table 1:** Characteristics of 284 traumatic cardiac arrest patients treated at a Swedish level 1 trauma centre between years 2011–2020

		Outcome at 30 days						
	**Total cohort**		**Dead**	**Alive**			**P value**	
	**(n = 284)**		**(n = 254)**	**(n = 30)**				
**Age (years)**								
Median [IQR]	38.0 [24.0–58.0]		38.0 [24.0–56.0]	43.5 [28.3–66.0]			0.24	
Missing	0 (0.0%)		0 (0.0%)	0 (0.0%)				
**Age groups (years)**								
15–24	74 (26.1%)		69 (27.2%)	5 (16.7%)			0.08	
25–34	48 (16.9%)		43 (16.9%)	5 (16.7%)				
35–49	58 (20.4%)		52 (20.5%)	6 (20%)				
50–64	47 (16.5%)		43 (16.9%)	4 (13.3%)				
65–79	46 (16.2%)		36 (14.2%)	10 (33.3%)				
80–94	11 (3.9%)		11 (4.3%)	0 (0.0%)				
**Gender**								
Male	233 (82.0%)		209 (82.3%)	24 (80.0%)			0.96	
Female	51 (18.0%)		45 (17.7%)	6 (20.0%)				
**Preinjury ASA class**								
1	171 (60.2%)		157 (61.8%)	14.0 (46.7%)			0.03	
2	44 (15.5%)		35 (13.8%)	9 (30.0%)				
3	37 (13.0%)		31 (12.2%)	6 (20.0%)				
4	2 (0.7%)		1 (0.4%)	1 (3.3%)				
Missing	30 (10.6%)		30 (11.8%)	0 (0.0%)				
**Dominant injury**								
Blunt	184 (64.8%)		163 (64.2%)	21 (70.0%)			0.67	
Penetrating	100 (35.2%)		91 (35.8%)	9 (30.0%)				
**Injury mechanism**								
Motor vehicle accident - non motorbike	32 (11.3%)		27 (10.6%)	5 (16.7%)			0.28	
Motor bike accident	17 (6.0%)		15 (5.9%)	2 (6.7%)				
Bike accident	8 (2.8%)		7 (2.8%)	1 (3.3%)				
Injured pedestrian	17 (6.0%)		14 (5.5%)	3 (10.0%)				
Other vehicle accident	4 (1.4%)		4 (1.6%)	0 (0.0%)				
Gunshot wound	44 (15.5%)		42 (16.5%)	2 (6.7%)				
Stab wound	56 (19.7%)		50 (19.7%)	6 (20.0%)				
Hit by blunt object	28 (9.9%)		26 (10.2%)	2 (6.7%)				
Same level fall	7 (2.5%)		4 (1.6%)	3 (10.0%)				
Fall from height	65 (22.9%)		60 (23.6%)	5 (16.7%)				
Explosion	1 (0.4%)		1 (0.4%)	0 (0.0%)				
Other	2 (0.7%)		2 (0.8%)	0 (0.0%)				
Missing	3 (1.1%)		2 (0.8%)	1 (3.3%)				
**Arrest rhythm**								
VT/VF	19.0 (6.7%)		14 (5.5%)	5 (16.7%)			0.09	
Asystole	112 (39.4%)		103 (40.6%)	9 (30.0%)				
PEA	70 (24.6%)		65 (25.6%)	5 (16.7%)				
Non shockable unknown rhythm	25 (8.8%)		22 (8.7%)	3 (10.0%)				
Missing	58 (20.4%)		50 (19.7%)	8 (26.7%)				
**Shockable rhythm**								
Yes	19 (6.7%)		8 (3.1%)	2 (6.7%)			0.04	
No	207 (72.9%)		6 (2.4%)	3 (10.0%)				
Missing	58 (20.4%)		50 (19.7%)	8 (26.7%)				
**ISS**								
Median [IQR]	38.0 [25.5–75.0]		41.0 [26–75.0]	26.0 [17.0–38.0]			< 0.001	
Missing	5 (1.8%)		5 (2.0%)	0 (0.0%)				
**NISS**								
Median [IQR]	50.0 [34.0–75.0]		57.0 [35.5–75.0]	41.0 [24.0–50.0]			< 0.001	
Missing	7 (2.5%)		6 (2.4%)	1 (3.3%)				
**Place of arrest**								
Out of hospital	256 (90.1%)		238 (93.7%)	18 (60.0%)			< 0.001	
In hospital	28 (9.9%)		16 (6.3%)	12 (40.0%)				

### Primary outcome

**Fig. 2:**
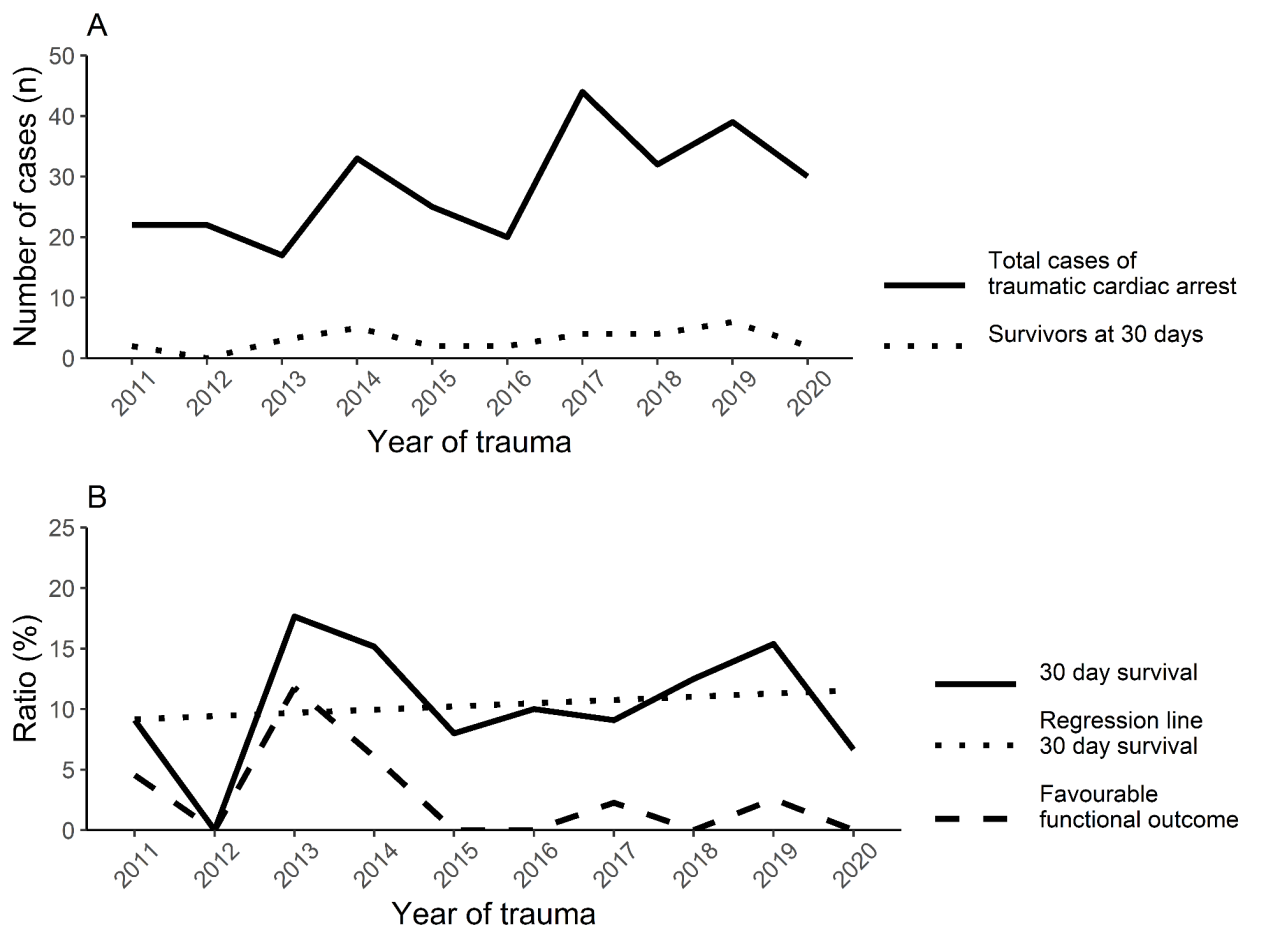
Temporal trends in traumatic cardiac arrest at a Swedish level 1 trauma centre during 2011–2020. (A) Caseload and survivors. (B) Total survivors and patients with favourable functional outcome as defined by Glasgow Outcome Scale score of 4–5. Regression line to evaluate trend in overall survival (P = 0.66)

A total of 30 patients (10.6%) survived to 30 days. Seven patients were assessed as GOS 4 and 23 patients as GOS 3 at discharge. No patients were discharged without disability. The survival rate was higher after in-hospital TCA (42.9%) as compared to pre-hospital TCA (7.0%) (P < 0.001). The survival rates varied between years, and we found no temporal trend (Fig. 2).

**Fig. 3:**
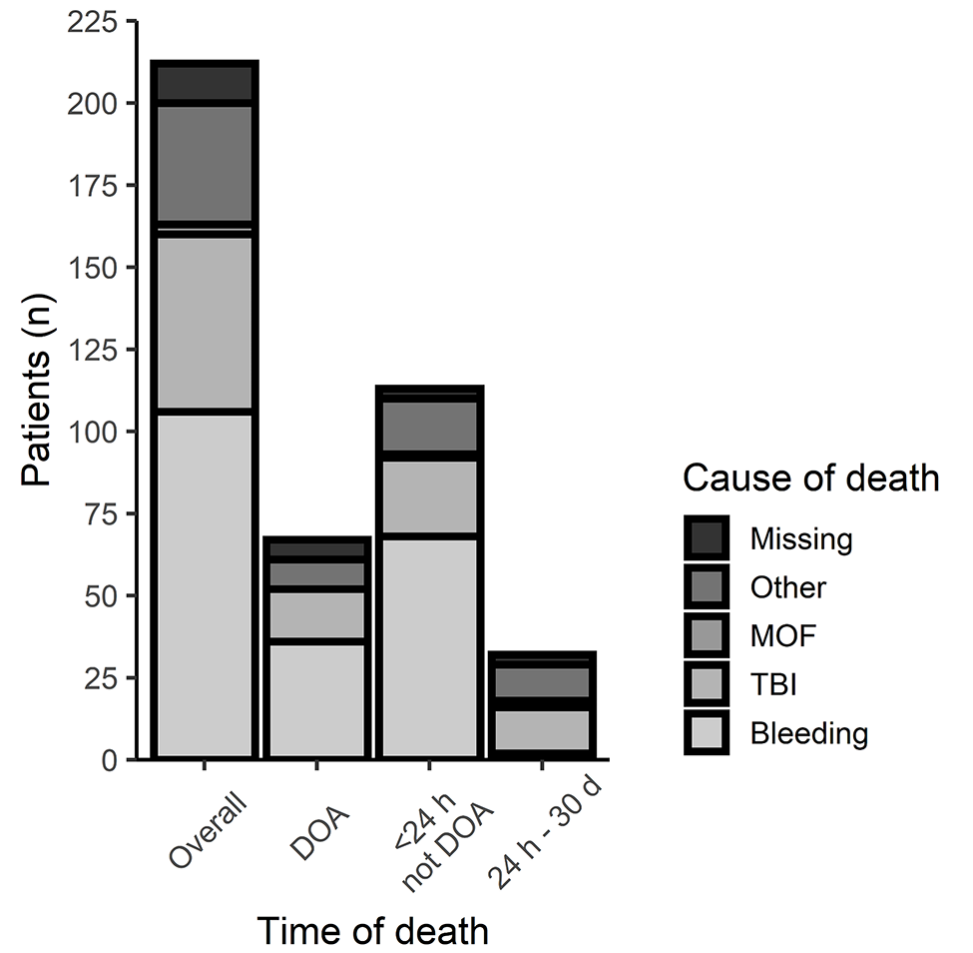
Summary of causes of mortality presented for 212 patients from year 2013 to 2020 grouped by time of death. MOF = Multi organ failure, TBI = Traumatic brain injury, DOA = Dead on arrival

Data on cause of death were only available from 2013 to 2020 for 212 TCA patients (Fig. 3.). Half of these died of bleeding. When death occurred within 24 h the proportion of deaths resulting from bleeding was even higher (60.2%). Between 24 h and 30 days traumatic brain injury (TBI) emerged as a more prevalent mortality cause (43.8%). On review, seven deaths were judged potentially preventable, of which five were considered due to bleeding, one because of TBI and the last cause was unspecified.

### Prehospital care

A majority (83.6%) of patients in the subset with out of hospital TCA (n = 256) were intubated and in 26.1% a thoracic decompression was performed in the prehospital setting. The median EMS response time was 9 min, median on scene time was 20 min and median transport time was 13 min and did not differ between groups (Table 2). Survivors compared to non-survivors received adrenaline (epinephrine) less frequently (16.7 vs. 60.1% (P < 0.001)) and in lower median amounts (0.0 (IQR 0.0–0.0) vs. 2.0 (IQR 0.0–4.0) mg (P = 0.002)) (Table 2).

**Table 2:** Prehospital interventions and dispatch times among the subset of 256 patients with out-of-hospital traumatic cardiac arrest

			Outcome at 30 days			
		**Total cohort**		**Dead**	**Alive**	**P value**
		(n = 256)		(n = 238)	(n = 18)	
**Bystander CPR**						
Yes		111 (43.4%)		102 (42.9%)	9 (50.0%)	0.93
No		88 (34.4%)		81 (34.0%)	7 (38.9%)	
EMS witnessed cardiac arrest		33 (12.9%)		31 (13.0%)	2 (11.1%)	
Missing		24 (9.4%)		24 (10.1%)	0 (0.0%)	
**Highest competence**						
Not attended by EMS		3 (1.2%)		3 (1.2%)	0 (0.0%)	0.97
Advanced life support by nurse		135 (52.7%)		125 (52.5%)	10 (55.6%)	
Advanced life support by physician		117 (45.7%)		109 (45.8%)	8 (44.4%)	
Missing		1 (0.4%)		1 (0.4%)	1 (0.4%)	
**EMS response time (min)**						
Median [IQR]		9.0 [6.0–13.0]		9.0 [6.0–13.0]	9.0 [6.0–13.0]	0.92
Missing		2 (0.8%)		2 (0.8%)	0 (0.0%)	
**On scene time (min)**						
Median [IQR]		20.0 [14.3–26.0]		19.5 [15.0–26.0]	21.5 [12.0-23.8]	0.69
Missing		2 (0.8%)		2 (0.8%)	0 (0%)	
**Transport time from scene to hospital (min)**						
Median [IQR]		13.0 [9.0–18.0]		13.0 [9.0–18.0]	14.5 [11.3–19.0]	0.31
Missing		2 (0.8%)		2 (0.8%)	0 (0.0%)	
**Time from dispatch to hospital (min)**						
Median [IQR]		44.0 [37.8–54.3]		44.0 [34.3–54.8]	46.0 [36.3–53.3]	0.79
Missing		0 (0.0%)		0 (0.0%)	0 (0.0%)	
**Intubation by EMS**						
Yes		212 (82.8%)		199 (83.6%)	13 (72.2%)	0.36
No		44 (17.2%)		39 (16.4%)	5 (27.8%)	
Missing		0 (0.0%)		0 (0.0%)	0 (0.0%)	
**Highest ETCO2 (kPa)**						
Median [IQR]		4.0 [2.2-6.0]		4.0 [1.9-6.0]	5.0 [4.1–6.1]	0.24
Missing		166 (64.8%)		153 (64.3%)	13 (72.2%)	
**Adrenaline (Epinephrine) administered**						
Yes		146 (57.0%)		143 (60.1%)	3 (16.7%)	< 0.001
No		75 (29.3%)		63 (26.5%)	12 (66.7%)	
Missing		35 (13.7%)		32 (13.4%)	3 (16.7%)	
**Amount of adrenaline (mg)**						
Median [IQR]		2.0 [0.0–4.0]		2.0 [0.0–4.0]	0.0 [0.0–0.0]	0.002
Missing		41 (16.0%)		38 (16.0%)	3 (16.7%)	
**Thoracic decompression by EMS**						
Yes		66 (25.8%)		62 (26.1%)	4 (22.2%)	0.94
No		190 (74.2%)		176 (73.9%)	14 (77.8%)	

### Intrahospital assessment and procedures

Survivors more commonly presented to the hospital with a pulse compared to non-survivors (70.0% (n = 21) vs. 21.7% (n = 55), P < 0.001). Among survivors of prehospital TCA (n = 18), 77.8% (n = 14) had regained spontaneous circulation when arriving in the trauma unit, 11.1% (n = 2) were in PEA and the remaining 11.1% (n = 2) had no rhythm documented.

A shockable rhythm was more frequently reported in survivors compared to non-survivors (16.7 vs. 5.5%, P = 0.04). Patients with asystole had a survival of 8.0%.

A pupillary response was more commonly elicited in survivors compared to non-survivors (56.7% vs. 9.8%, P < 0.001).

**Fig. 4:**
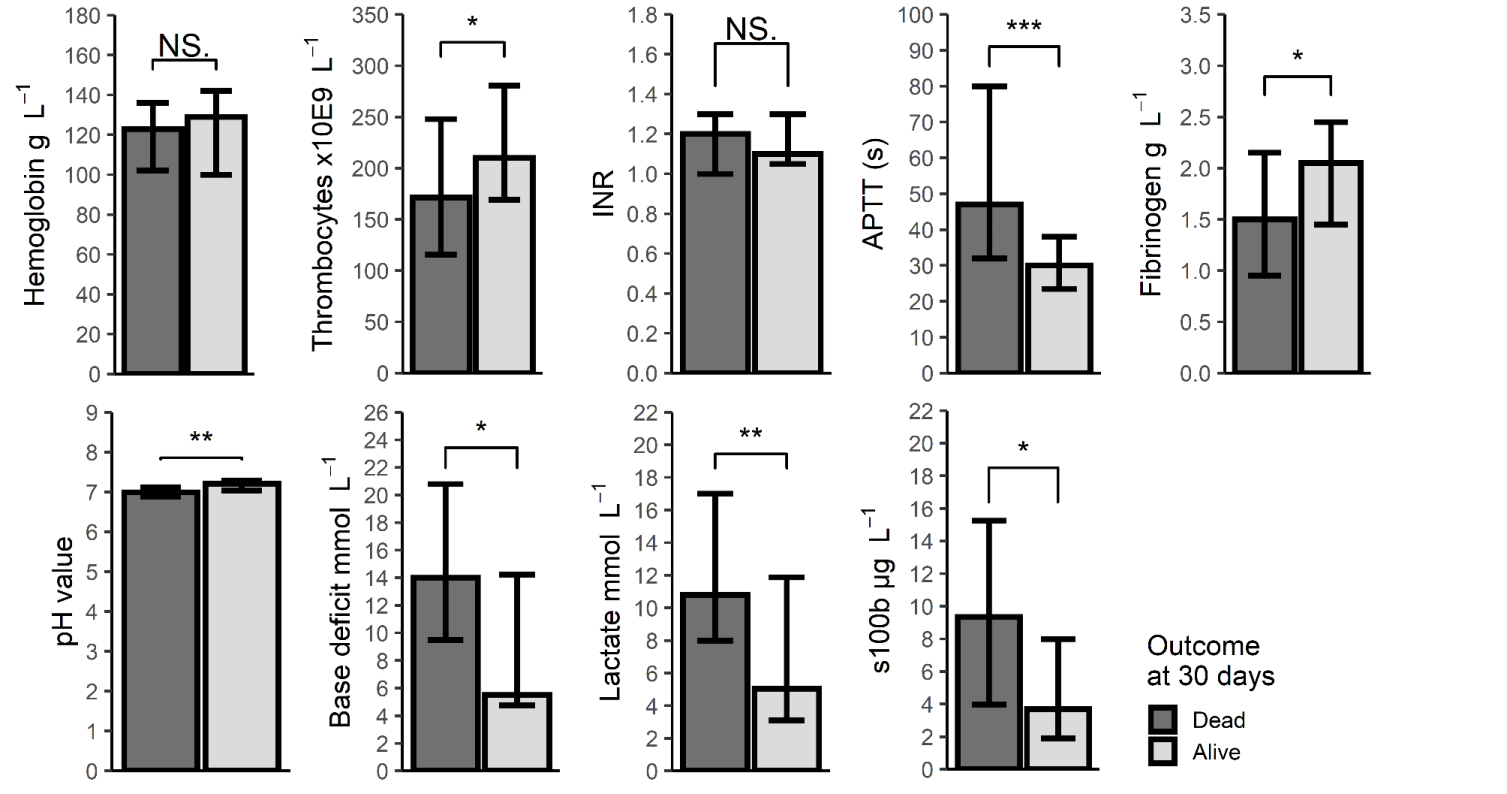
Lab values at hospital admission. Data are presented as median and interquartile range. Difference between groups examined with Wilcoxon rank sum test. *=P < 0.05, **= P < 0.01, ***= P < 0.001, NS = Nonsignificant at 0.05 level

Survivors exhibited higher platelet counts, higher fibrinogen, shorter activated partial thromboplastin time (APTT), lower lactate, lower base deficit, higher pH value and a lower S100b in comparison to non-survivors (Fig. 4). The highest measured lactate in a survivor was 20 mmol L^− 1^. Concerning lab values there were variable amounts of missing data (Supplementary Table 2).

### Resuscitative thoracotomy

Altogether, 101 TCA patients (36%) underwent resuscitative thoracotomy. Among these, 12 patients (11.9%) survived with a GOS score at hospital discharge of 3 (n = 8) or 4 (n = 4).

A tamponade was released in two of the survivors (16.7%) and 12 of the non survivors (13.5%). In two survivors, during one case of extremity bleed and another of abdominal bleed, a thoracotomy was made solely to occlude the aorta.

**Fig. 5:**
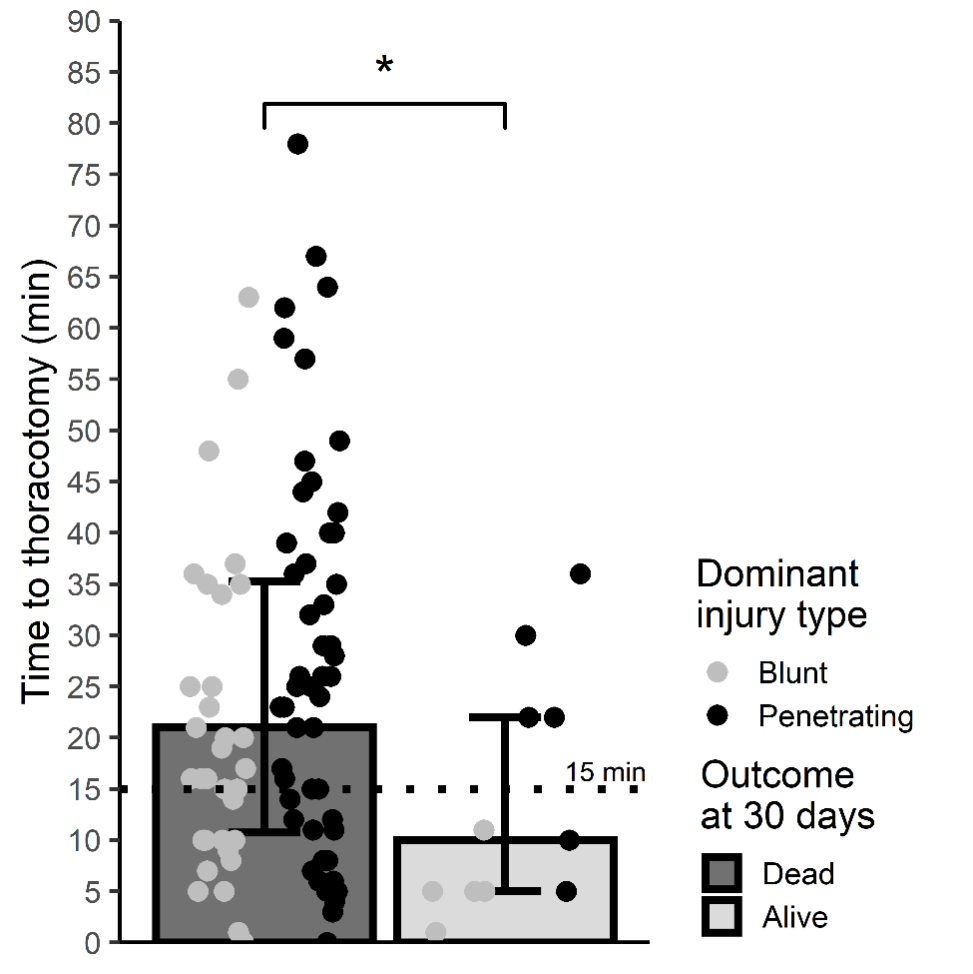
Time from last vital sign to start of thoracotomy. Dotted line marks 15 min corresponding to upper recommended limit for resuscitative thoracotomy in European Resuscitation Council’s current guidelines^4^. Data presented as median and interquartile range. Scatterplot superimposed to present individual cases. Comparison between groups with Wilcoxon rank sum test. *=P < 0.05

Patients arresting after arrival had a higher survival after thoracotomy compared to patients presenting in arrest (42.1 vs. 4.9%, P < 0.001). Median time from last sign of life to thoracotomy was shorter for in-hospital TCA compared to prehospital TCA (9.5 (IQR 5-14.75) vs. 23 (IQR 14–36) minutes, P < 0.001). For prehospital TCA, the median time from hospital arrival to start of thoracotomy was 4 (IQR 3–10) minutes. Blunt and penetrating TCAs had a comparable survival after thoracotomy (12.5 and 11.5%, P = 1), however median time to resuscitative thoracotomy from last sign of life was shorter in blunt than penetrating trauma (15.5 (IQR 8.3–24.5) vs. 25.0 (IQR 12.0–37.0) minutes, P = 0.01). Median time from latest sign of life to resuscitative thoracotomy was shorter in survivors compared to non-survivors (10 (IQR 5.0–22.0) vs. 21 (IQR 10.8–35.3) minutes (P = 0.03) (Fig. 5).

Resuscitative thoracotomy was in 58 patients performed later than the suggested 15 min since loss of vital signs (Fig. 5). Of those, four patients survived of whom all had suffered penetrating injuries. Three survivors had arrested before hospital arrival and one during initial resuscitation. One survivor with PEA and one with unknown rhythm had recordings of visible cardiac contractions upon chest opening. Another survivor who was in asystole had a pericardial tamponade relieved. The last survivor who had the longest arrest duration of 36 min before thoracotomy was also in PEA. One survivor was discharged with GOS 4 and the remaining three with GOS 3.

## Discussion

This single-centre retrospective cohort study demonstrated a 10.6% 30-day survival for TCA between years 2011–2020. Most deaths were caused by bleeding and non-survivors were found more coagulopathic. We identified nine survivors with asystole as arrest rhythm. Survival after resuscitative thoracotomy was 11.9% and four among these 12 survivors breached the upper time limit of 15 min stipulated in current European guidelines [4].

The demonstrated survival in our series is largely in keeping with recent evidence describing survival ranging from 2.4 to 18.4% [3, 6–17]. However, direct comparison with previous outcome studies is difficult due to a wide variation in factors such as inclusion criteria, geographic settings and development of health care systems. The only prior survey of TCA set in Sweden quoted an overall survival of 3.7% for out-of-hospital TCA between 1990 and 2015 [3] as compared to 7% in the subset of prehospital TCA in our material. In relation to data from the Swedish Cardiac Arrest Registry, that mainly reports on medical arrests, we found a moderately lower overall survival for out-of-hospital TCA (7 vs. 11%) but a slightly better survival for in-hospital TCA (42.9% vs. 35%) during the same ten years [29].

Favourable outcome among survivors was recorded in 23% in our study which was merely about half of the 56% in a recent review [30] and although four patients survived after a late thoracotomy only one had a favourable outcome. The rate of severe disability in survivors is rather high. However, this finding must be interpreted in the light of that GOS score at discharge in multi-trauma often is impacted by concomitant traumatic injuries affecting whole body function, hence not purely reflecting cerebral performance. Later improvement of GOS may have occurred but unfortunately long-term data were not available.

Bleeding was the most predominant early death cause, surpassed by TBI after 24 h of care in line with previous literature [6, 31]. Most preventable deaths were also judged to be caused by bleeding. We furthermore found non-survivors to have more affected coagulation tests. Trauma-induced coagulopathy correlates to an increased mortality [32], especially in severely injured patients [33], and can occur in TBI even in the absence of major haemorrhage [34]. This emphasises the need to swiftly control bleeding and initiate massive transfusion as stressed in present guidelines [4].

In our survey, survivors with each arrest rhythm were reported including 9 patients with asystole. Primary rhythm has been proposed as a triage tool where resuscitation is withheld in asystolic TCA [35–37]. However, contrasting studies have shown that survival in asystole is possible [7] even with complete neurological recovery [38]. These findings suggest a poor prognostic power of absent electrical activity as a stand-alone criterion to stop resuscitation. Moreover, we noticed that survivors more often recorded a shockable rhythm which in a review was shown to be prognostic for survival [30]. Though, it is still possible that in cases with shockable rhythm, the cardiac arrest preceded the trauma and was actually medical in origin.

In the subgroup of prehospital TCA, non-survivors more often received adrenaline and in higher doses. In a recent meta-analysis it was concluded that adrenaline use in TCA predicts return of spontaneous circulation (ROSC) while decreasing odds of survival [30]. Adrenaline is known to impair cerebral micro-circulation and increases myocardial oxygen demand [39] which in a hypovolemic low-flow state might worsen outcome. Additionally, it is also plausible that this correlation is confounded by adrenaline acting as a proxy for longer arrest times.

In our study, survival after resuscitative thoracotomy was 11.9% which is slightly higher than the 7.8% in a recent review [18]. This despite 57% of thoracotomies being carried out later than 15 min from loss of vital signs [4]. Nevertheless, we found that survivors had shorter times from arrest to resuscitative thoracotomy, underscoring the need for a system facilitating fast transport times and instant access to surgical competence [25]. This has previously been established in a military context where shorter time intervals (6.2 vs. 17.7 min for survivors and non-survivors respectively) yielded an even higher overall survival of 21.5% [20].

Interestingly, we found a similar survival after resuscitative thoracotomy between blunt and penetrating injuries which contradicts previous studies reporting a poorer outcome after blunt trauma [36, 40, 41]. This may be explained by the shorter time to thoracotomy in blunt compared to penetrating victims in our material, conceivably influenced by local protocols recommending resuscitative thoracotomy within 10 min in blunt but up to 15 min for penetrating trauma.

Surprisingly, four of the 12 survivors after resuscitative thoracotomy breached the 15-minute restriction set in recent European guidelines [4]. When the futility of resuscitative thoracotomy in TCA was previously investigated, no survivors were found with prehospital CPR-times exceeding 15 min [42]. It is certainly pertinent to separate potential survivors not to misdirect unwarranted resources. Firstly, all four survivors suffered penetrating trauma known to have better outcome after thoracotomy [36, 40, 41]. Moreover, focused assessed sonography in trauma (FAST) has been showing promising prognostic value in TCA with a high sensitivity for survival [30, 43]. The two survivors with cardiac motion, possibly in a low flow state rather than true arrest, would expectedly have been identified using FAST. Specifically, by using the protocol suggested by Inaba and co-workers, where absence of both contractions and pericardial effusion on FAST carried a 0% survival chance, thoracotomy would have been regarded futile in only 1/4 survivors [44]. An upper boundary of 15 min for resuscitative efforts in TCA has also been challenged in four previous papers. Cumulatively, 16% of survivors in these studies were in violation of the 15-minute limit [4]; 7/68 in London [17], 1/4 in Victoria [45], 3/14 in Seattle [7] and 4/6 in Taiwan [46]. These combined observations mandate caution in applying this time-related criterion.

### Strengths and limitations

The key strength of this study is that we report on both trauma-related and arrest specific variables with complete coverage of outcome parameters. Limitations of this study include those inherent to a retrospective database review. In some areas the amount of missing data was not minor, especially regarding lab values, and these results need to be interpreted with caution. In addition, we lack information on the proportion of patients where resuscitation was terminated on scene.

## Conclusion

Our data support that survival after TCA is possible and that aggressive care is justified, particularly directed at managing bleeding. Determining futility in TCA is complex and although current treatment algorithms mostly perform adequately, our study demonstrates exceptions where patients survived outside of guidelines.

## Electronic supplementary material

Below is the link to the electronic supplementary material.

Supplementary Material 1

Supplementary Material 2

## Data Availability

The datasets used and/or analysed during the current study are available from the authors on reasonable request and in compliance with the General data protection regulation and Swedish legislation.

## Abbreviations

***TCA***: Traumatic cardiac arrest.
***EMS***: Emergency Medical Services.
***CPR***: Cardiopulmonary resuscitation.
***ISS***: Injury Severity Score.
***GOS***: Glasgow Outcome Scale.
***PEA***: Pulseless electric activity.
***IQR***: Interquartile range.
***TBI***: Traumatic brain injury.
***FAST***: Focused assessed sonography in trauma.
