# Supplementary material for: Characteristics and outcome of traumatic cardiac arrest at a level 1 trauma centre over 10 years in Sweden
Source: Scand J Trauma Resusc Emerg Med. 2022 Oct 17;30:54. doi: 10.1186/s13049-022-01039-9 (PMC9575295; doi:10.1186/s13049-022-01039-9)
Supplement: Supplementary file 1 — Supplementary Material 1 [file 13049_2022_1039_MOESM1_ESM.docx]

| **Supplementary table 1. Variable list** | | | | |
| --- | --- | --- | --- | --- |
| **Variable** | **Description** | **Values** | **Data available** | **Source of information** |
| **Age** | Age of the person in the study | numeric | 2011-2020 | SweTrau |
| **Gender** | Gender of the person in the study | binary | 2011-2020 | SweTrau |
| **Dominant injury** | The dominant injury of the person in the study | numeric | 2011-2020 | SweTrau |
| **Mechanism of injury** | Mechanism of the injury that occured | numeric | 2011-2020 | SweTrau |
| **ASA** | ASA (American Society of Anaesthesiology) class before the trauma | numeric | 2011-2020 | SweTrau, TakeCare |
| **Ventilator days** | How many days the person spent in a ventialtor | numeric | 2011-2020 | SweTrau |
| **LOS** | How many days the person spent in hospital | numeric | 2011-2020 | SweTrau |
| **GOS** | The Glasgow Outcome scale at discharge from hospital | numeric | 2011-2020 | SweTrau |
| **ISS** | ISS is a score that attempts to standardize the severity of injuries sustained during a trauma. | numeric | 2011-2020 | SweTrau |
| **NISS** | New Injury Severity **Score** (**NISS**) | numeric | 2011-2020 | SweTrau |
| **30-day survival** | If the person survived for 30 days or not | numeric | 2011-2020 | SweTrau |
| **Cause of death** | The cause of death decided during peer review of charts and coroner report. | numeric | 2013-2020 | SweTrau |
| **Preventible** | If the death was considered preventable. | binary | 2013-2020 | SweTrau |
| **Survival 24 h** | If the person survived 24 h | binary | 2011-2020 | TakeCare |
| **DOA** | If the person arrival at the ED was dead and nothing was to be done | binary | 2011-2020 | TakeCare |
| **Witnessed trauma** | If the trauma was witnessed | binary | 2011-2020 | FRAPP, TakeCare |
| **Bystander CPR** | If non-EMS performed CPR in the field | binary | 2011-2020 | FRAPP, TakeCare |
| **Date time trauma** | The time when the trauma occured | not applicable | 2011-2020 | SweTrau |
| **Date time arrival scene** | The time then when the EMS arrived at the scene where the trauma occured | not applicable | 2011-2020 | SweTrau |
| **Date time leave scene** | The time when the EMS leaved the scene where the trauma occurred | not applicable | 2011-2020 | SweTrau |
| **Date time at hospital** | Time when when the person arrived at the hospital | not applicable | 2011-2020 | SweTrau |
| **EMS response time** | How long it took the EMS personnel from the alarm to respond at scene | numeric | 2011-2020 | SweTrau |
| **EMS depart** | How long it took the EMS personnel from arrival to depart from scene | numeric | 2011-2020 | SweTrau |
| **EMS arrival hospital** | How long it took the EMS personnel from arrival at scene to arrival to hospital | numeric | 2011-2020 | SweTrau |
| **EMS transport time** | How long it took the EMS personnel from scene to hospital | numeric | 2011-2020 | SweTrau |
| **Airway management by EMS** | Wheter advanced airway management was performed by EMS | numeric | 2011-2020 | SweTrau |
| **Adrenaline** | If adrenaline were given prehospital or not and in what amount | binary/numeric | 2011-2020 | FRAPP |
| **ETCO2** | Highest measured endtidal carbon dioxide in the prehospital setting | numeric | 2011-2020 | FRAPP |
| **Prehospital intubation** | If the person were intubated in the prehospital setting | binary | 2011-2020 | SweTrau |
| **Airway trauma unit** | How the airway was handled in the trauma unit | numeric | 2011-2020 | SweTrau |
| **Reactive pupil** | If any pupil was reaktice in the ED | binary | 2011-2020 | TakeCare |
| **Blood test** | Time when first blood tests were drawn | numeric | 2011-2020 | TakeCare |
| **Haemoglobin** | Hemoglobin level sampled in the trauma unit | numeric | 2011-2020 | TakeCare |
| **Thrombocytes** | Thrombocyte count sampled in the trauma unit | numeric | 2011-2020 | TakeCare |
| **APTT** | APTT sampled in the trauma unit | numeric | 2011-2020 | TakeCare |
| **INR** | The INR value in the trauma unit | numeric | 2011-2020 | SweTrau |
| **Fibrinogen** | Fibrinogen sampled in the trauma unit | numeric | 2011-2020 | TakeCare |
| **pH** | pH sampled in the trauma unit | numeric | 2011-2020 | TakeCare |
| **Base excess** | Base excess sampled in the trauma unit | numeric | 2011-2020 | TakeCare |
| **Lactate** | Lactate sampled in the trauma unit | numeric | 2011-2020 | TakeCare |
| **Arrest rhythm** | First recorded rhythm during cardiac arrest | binary | 2011-2020 | TakeCare |
| **Thoracotomy start** | The time when the thoracotomy was started | numeric | 2011-2020 | SweTrau |
| **Last sign of life** | When last signs of life were recorded either through time stated or when interval given correlated to time-stamped event in the registry. | numeric | 2011-2020 | FRAPP, TakeCare |
| **TakeCare:** Electronic chart system containing medical records. Used at at Karolinska university hospital from before the start of 2011.  **FRAPP:** Electronic records of prehospital charts, including times, vital parameters and interventions.  **SweTrau:** The Swedish national trauma registry | | | | |
|  | | | | |
|  | | | |  |
