# Supplementary material for: Characteristics and outcome of traumatic cardiac arrest at a level 1 trauma centre over 10 years in Sweden
Source: Scand J Trauma Resusc Emerg Med. 2022 Oct 17;30:54. doi: 10.1186/s13049-022-01039-9 (PMC9575295; doi:10.1186/s13049-022-01039-9)
Supplement: Supplementary file 2 — Supplementary Material 2 [file 13049_2022_1039_MOESM2_ESM.docx]

| **Supplementary table 2. In-hospital investigations in 248 traumatic cardiac arrest patients.** | | | | | | | |
| --- | --- | --- | --- | --- | --- | --- | --- |
|  |  | | Outcome at 30 days | | | |  |
|  | | **Total cohort** | | **Dead** | **Alive** | **P-value** | |
|  | | (n=284) | | (n=254) | (n=30) |  | |
| **Reactive pupil** | |  | |  |  |  | |
| Yes | | 42 (14.8%) | | 25 (9.8%) | 17 (56.7%) | <0.001 | |
| No | | 176 (62.0%) | | 17 (66.9%) | 6 (20.0%) |  | |
| Missing | | 66 (23.2%) | | 59 (23.2%) | 7 (23.3%) |  | |
| **Haemoglobin (g L^-1^)** | |  | |  |  |  | |
| Median [IQR] | | 124.0 [102.0-138.0] | | 123.0 [112.0-136.0] | 129.0 [100.0-142.0] | 0.49 | |
| Missing | | 148 (52.1%) | | 147 (57.9%) | 1 (3.3%) |  | |
| **Fibrinogen (g L^-1^)** | |  | |  |  |  | |
| Median [IQR] | | 1.6 [1.0-2.2] | | 1.5 [1-2.2] | 2.1 [1.4-2.3] | 0.02 | |
| Missing | | 159 (56.0%) | | 155 (61.0%) | 4 (13.3%) |  | |
| **APTT (seconds)** | |  | |  |  |  | |
| Median [IQR] | | 43.0 [28.3-73] | | 47.0 [32.0-80.0] | 30.0 [23.5-38.0] | <0.001 | |
| Missing | | 158 (55.6%) | | 155 (61.0%) | 3 (10.0%) |  | |
| **INR** | |  | |  |  |  | |
| Median [IQR] | | 1.2 [1.0-1.3] | | 1.2 [1.1-1.3] | 1.1 [1.1-1.3] |  | |
| Missing | | 155 (54.6%) | | 152 (59.8 %) | 3 (10.0%) |  | |
| **Thrombocytes**  **(x10E9 L^-1^)** | |  | |  |  |  | |
| Median [IQR] | | 185.0 [128.8-250.5] | | 172.0 [115.8-248.0] | 210.0 [169.2-  280.5] | 0.05 | |
| Missing | | 158 (55.6%) | | 154 (60.6%) | 4 (13.3%) |  | |
| **S100b (µg L^-1^)** | |  | |  |  |  | |
| Median [IQR] | | 7.4 [2.9-13.5] | | 9.4 [4.0-15.3] | 3.7 [1.9-8.0] | 0.009 | |
| Missing | | 197 (69.4%) | | 186 (72.7%) | 11 (36.7%) |  | |
| **pH value** | |  | |  |  |  | |
| Median [IQR] | | 7.0 [6.9-7.2] | | 7.0 [6.9-7.1] | 7.2 [7.0-7.3] | 0.005 | |
| Missing | | 233 (82.0%) | | 219 (86.2%) | 14 (46.7%) |  | |
| **Base deficit**  **(mmol L^-1^)** | |  | |  |  |  | |
| Median [IQR] | | 12.0 [6.5-19.0] | | 14.0 [9.5-20.8] | 5.5 [4.8-14.3] | 0.03 | |
| Missing | | 237 (83.5%) | | 223 (87.8%) | 14 (46.7%) |  | |
| **Lactate (mmol L^-1^)** | |  | |  |  |  | |
| Median [IQR] | | 9.7 [6.0-15.1] | | 10.8 [8.0-17.0] | 5.1 [3.1-11.9] | 0.001 | |
| Missing | | 207 (72.9%) | | 197 (77.6%) | 10 (33.3%) |  | |
| All lab values were sampled in the trauma unit. Differences between survivors and non-survivors at 30 days were calculated with Wilcoxon signed rank test for numerical values and Chi-square test of independence for categorical values. | | | | | | | |
